# Phylogenetic diversity and functional gene patterns of sulfur-oxidizing subseafloor *Epsilonproteobacteria* in diffuse hydrothermal vent fluids

**DOI:** 10.3389/fmicb.2013.00185

**Published:** 2013-07-08

**Authors:** Nancy H. Akerman, David A. Butterfield, Julie A. Huber

**Affiliations:** ^1^Josephine Bay Paul Center for Comparative Molecular Biology and Evolution, Marine Biological LaboratoryWoods Hole, MA, USA; ^2^Joint Institute for the Study of the Atmosphere and Ocean, University of Washington and NOAA Pacific Marine Environmental LabSeattle, WA, USA

**Keywords:** sulfur oxidation, hydrothermal vent microbiology, 16S rRNA, functional genes, subseafloor, *Epsilonproteobacteria*

## Abstract

Microorganisms throughout the dark ocean use reduced sulfur compounds for chemolithoautotrophy. In many deep-sea hydrothermal vents, sulfide oxidation is quantitatively the most important chemical energy source for microbial metabolism both at and beneath the seafloor. In this study, the presence and activity of vent endemic *Epsilonproteobacteria* was examined in six low-temperature diffuse vents over a range of geochemical gradients from Axial Seamount, a deep-sea volcano in the Northeast Pacific. PCR primers were developed and applied to target the sulfur oxidation *soxB* gene of *Epsilonproteobacteria*. *soxB* genes belonging to the genera *Sulfurimonas* and *Sulfurovum* are both present and expressed at most diffuse vent sites, but not in background seawater. Although *Sulfurovum*-like *soxB* genes were detected in all fluid samples, the RNA profiles were nearly identical among the vents and suggest that *Sulfurimonas*-like species are the primary *Epsilonproteobacteria* responsible for actively oxidizing sulfur via the Sox pathway at each vent. Community patterns of subseafloor *Epsilonproteobacteria* 16S rRNA genes were best matched to methane concentrations in vent fluids, as well as individual vent locations, indicating that both geochemistry and geographical isolation play a role in structuring subseafloor microbial populations. The data show that in the subseafloor at Axial Seamount, *Epsilonproteobacteria* are expressing the *soxB* gene and that microbial patterns in community distribution are linked to both vent location and chemistry.

## Introduction

Sulfur is an abundant, multi-valent element in deep-sea hydrothermal vent systems. Large metal sulfide chimneys and biofilms containing filamentous sulfur are often present, and hydrogen sulfide concentrations are typically in the millimolar range (Sievert et al., 2008a). The high concentrations of hydrogen sulfide (H_2_S) at deep-sea vents are produced mainly via high-temperature seawater-rock interactions in the subseafloor hydrothermal reaction zone (Jannasch and Mottl, 1985). Other partially reduced sulfur compounds, such as thiosulfate, polysulfide, and elemental sulfur, are generated when hydrothermal vent fluids mix with oxygenated seawater (Yamamoto and Takai, 2011). At deep-sea hydrothermal vent systems, the microbially-mediated oxidation of reduced sulfur compounds is a key chemolithotrophic process that provides a substantial primary energy source for higher organisms (Jannasch and Mottl, 1985; Sievert et al., 2008a). Thermodynamic modeling studies show that sulfide oxidation is the major energy source at most non-peridotite, basalt-hosted deep-sea hydrothermal vent systems (Amend et al., 2011), including Axial Seamount on the Juan de Fuca Ridge (Butterfield et al., 2004). Microbial sulfate reduction also occurs across a range of temperatures in hydrothermal discharge areas (Bonch-Osmolovskaya et al., 2011). Even away from hydrothermal vents, it is clear that reduced inorganic sulfur compounds play an important role in chemolithoautotrophic metabolism throughout the dark ocean (Walsh et al., 2009; Orcutt et al., 2011; Swan et al., 2011).

Many bacteria and some archaea have the capability of oxidizing sulfur or sulfide to sulfate. In bacteria, there are two different sulfur oxidation pathways: the reverse sulfate reduction pathway, which uses the Dsr, Apr, or Sat enzymes (Kappler and Dahl, 2001), and the sulfur oxidation Sox multienzyme system (Friedrich et al., 2001, 2005). The Sox system contains four protein components, SoxYZ, SoxXA, SoxB, and SoxCD, for the complete oxidation of sulfide and thiosulfate to sulfate (Friedrich et al., 2005). The SoxB protein, encoded by the *soxB* single copy gene, has been identified as the sulfate thiol esterase in the Sox system, and the *soxB* gene is used as a marker gene to survey sulfur oxidizing bacteria (Friedrich et al., 2005). Homologous proteins to SoxB have not been found in the domain Archaea (Friedrich et al., 2001). Polymerase chain reaction (PCR) primers targeting the *soxB* gene have previously been developed; these sets are based on *Chlorobia* and *Alphaproteobacteria* sequences and were refined using additional *Alphaproteobacteria* and *Aquificales* sequences (Petri et al., 2001). The primers have been extensively tested and found to amplify the *soxB* gene from mostly photo- and chemotrophic sulfur oxidizing species, including the *Gammaproteobacteria* (Meyer et al., 2007), with additional examples from the *Epsilonproteobacteria* (Hügler et al., 2010). However, some of the primer sets developed by Petri et al. (2001) are degenerate primers that result in multiple PCR amplicons of approximately the same size, which can give ambiguous sequencing results, and some key phylogenetic groups may also be missed when applied to diverse habitats (Petri et al., 2001; Meyer et al., 2007; Headd and Engel, 2013).

The purpose of this study was to design and test *soxB* PCR primers specific to the *Epsilonproteobacteria* to evaluate the presence, diversity, and gene expression of sulfur-oxidizing *Epsilonproteobacteria* at deep-sea hydrothermal vent systems. Chemolithoautotrophic *Epsilonproteobacteria* are often the most abundant group of bacteria detected in both the free-living and symbiotic microbial communities at deep-sea vents (e.g., Huber et al., 2003; Nakagawa et al., 2007; Nakagawa and Takai, 2008; Sievert et al., 2008a). Epsilonproteobacterial isolates from vents include *Sulfurimonas autotrophica* DSM16294, *Sulfurovum* sp. NBC37-1, and *Nitratiruptor* sp. SB155-2. All recently had their genomes sequenced (Nakagawa et al., 2007; Sikorski et al., 2010), along with a closely related coastal wetland isolate *S. denitrificans* DSM1251 (Sievert et al., 2008b), and all have the capability of oxidizing reduced sulfur compounds using the sulfur oxidation Sox multienzyme system (Yamamoto and Takai, 2011). This new genomic information was used to develop and test *soxB* gene PCR primers specific to the *Epsilonproteobacteria* at diffuse vents from Axial Seamount, where *Epsilonproteobacteria* are known to dominate many low-temperature vent fluids (Huber et al., 2003; Sogin et al., 2006; Huber et al., 2007; Opatkiewicz et al., 2009). After primer optimization and testing, six low-temperature diffuse hydrothermal vent samples that span a range of temperature, pH, sulfide, and geological settings were screened using both 16S rRNA 454 pyrosequencing and the new *soxB* gene primers to evaluate the presence, diversity, and gene expression of sulfur-oxidizing *Epsilonproteobacteria* at deep-sea hydrothermal vent systems.

## Experimental procedures

### Sample site

Axial Seamount (46°55′ N; 130°00′ W) is an active underwater volcano located approximately 250 nautical miles west of the Oregon/Washington coast at the intersection of the Juan de Fuca Ridge and the Cobb-Eickelberg Seamount Chain. The caldera of Axial Seamount is oriented northwest-to-southeast and is 3 × 8 km, with hydrothermal vent fields associated with the north and south rift zones and near the caldera boundary fault in the southern half of the caldera (Embley et al., 1990; Butterfield et al., 2004). All samples in this study were collected from hydrothermal sites along the southern caldera boundary fault.

### Sample collection

Hydrothermal fluid samples were collected in August and September 2010 using the ROV *Jason II* and the Hydrothermal Fluid and Particulate Sampler (HFPS) (Butterfield et al., 2004). The titanium intake nozzle with in-line temperature probe was inserted into the vents, with the tip generally penetrating into the seafloor. Filtered and unfiltered fluids were sampled after a steady in-line temperature was found and the sampling pump was then turned on to collect fluids at the rate of ~150 ml/min. Temperature was monitored and recorded throughout sampling and the maximum and average temperatures for the samples are reported (Table 1). Three liters of fluid was filtered onto Sterivex filters (Millipore) or 47-mm flat filters (Millipore) for DNA analysis and RNA analysis, respectively. For the 47 mm filters, a McLane “pancake” style filter assembly was used and filled with RNALater (Life Technologies), so that immediately after filtration, RNALater diffused over the filter surface and remained until ROV recovery (Cowen et al., 2012). A background seawater sample outside of the Axial Seamount caldera was collected via CTD at ~1500 m depth, and ~3 L of seawater was filtered onto a flat filter on board the ship. Sterivex filters were flooded with RNALater, sealed with Male/Female Luer Caps, and stored in 50-mL sterile Falcon tubes, while flat filters were stored in 5-mL sterile plastic tubes filled with RNALater. All filters were stored at 4°C for 24 h before being stored at −80°C until nucleic acid extraction in the laboratory. Fluid samples for chemistry were analyzed shipboard for pH, alkalinity, hydrogen sulfide, ammonia, methane and hydrogen following the methods of Butterfield et al. (2004). Major cations and anions were analyzed by ion chromatography and iron and manganese were analyzed by atomic absorption and/or ICP-MS on shore. Fluid samples were preserved in formaldehyde for cell enumeration using epifluorescence microscopy with DAPI as described previously (Huber et al., 2002).

**Table 1:** **Characteristics of vent fluid and seawater samples**.

	**Gollum**	**Marker 33**	**Marker 113**	**Pompeii**	**Escargot**	**9 m**	**Seawater**
Vent type	Basalt	Basalt	Basalt	Sulfide	Sulfide	Sulfide	
Cells/ml	5.1 × 10^5^	6.3 × 10^4^	6.7 × 10^5^	4.8 × 10^5^	3.8 × 10^5^	3.6 × 10^5^	1.8 × 10^4^
T_max_, °C	22.3	39.0	29.1	33.8	22.7	51.3	2
T_avg_, °C	21.4	38.3	27.9	31.1	20.6	49.7	2
pH	5.7	5.5	6.0	5.4	5.0	4.8	7.8
Avg Alk, Meq/kg	2.77	1.62	2.46	2.21	2.32	2.03	2.43
Avg Si, μmol/kg	815	2857	326	1568	1575	2169	155
Mg, mmol/kg	50.7	41.8	50.4	46.7	48.1	44.4	52.9
% Seawater^a^	96	79	95	88	91	84	100
H_2_S, μM	84	929	748	452	554	1626	0
NH_3_, μM	5.19	6.05	4.68	3.74	4.95	10.64	<0.6
H_2_S/Heat^b^	1	6.3	6.9	4	6.6	8.6	0
H_2_/Heat^b^	0.00	0.12	0.00	0.17	0.10	0.29	0
CH_4_/Heat^b^	0.09	0.13	0.38	0.05	0.11	0.06	0
Fe/Heat^b^	0.01	0.02	0.01	0.03	0.07	0.36	0
Fe/Mn	0.03	0.03	1.52	0.17	0.35	1.36	0
Mn/Heat^b^	0.21	0.59	0.01	0.18	0.21	0.26	0

a % Seawater calculated as Mg_vf_/Mg_sw_ × 100. Subscripts: vf, vent fluid; sw, seawater.

b Ratios to heat, reported in nmol/J, calculated as [C_vf_ − C_sw_]/[(T_vf_ − T_sw_) × C_p_] where C_p_ is the heat capacity of water (4.15 J g^−1^°C^−1^ for the temperature and pressure in this study).

### DNA extraction

Genomic DNA was extracted from Sterivex filters following the procedure outlined in Sogin et al. (2006) and Huber et al. (2002), with the following minor modifications. RNALater was removed from the filter via pushing the liquid out with a 3 ml syringe. 1.85 mL of DNA extraction buffer (0.1 M Tris-HCl [pH 8], 0.1 M Na_2_-EDTA [pH 8], 0.1 M NaH_2_PO_4_ [pH 8], 1.5 M NaCl, and 1% cetyltrimethlammonium bromide) was then added to the filter. Sterivex filters were then re-capped and the extraction method of Huber et al. (2002) with modifications in Sogin et al. (2006) followed. Successful DNA extraction was verified via PCR using bacterial 16S primers 8F (5′-AGA GTT TGA TCC TGG CTC AG-3′) and 1492R (5′-GGT TAC CTT GTT ACG ACT T-3′) and visualized under UV light on a 1% agarose gel stained with ethidium bromide.

### RNA extraction and reverse transcription PCR

RNA filters were allowed to thaw at room temperature in their 5-mL storage tubes. Total RNA was extracted using the RNAqueous-4PCR kit (Ambion), with the following adaptations. First, RNALater was discarded from the 5-mL tube, and 500 μ l of lysis/binding solution was added directly to the tube. Tubes were vortexed at high speed for ~2 min. The lysis/binding solution was removed from the tube and placed in a 2-mL collection tube. An additional 500 μ l of lysis/binding solution was added to the 5-mL tube, and the vortex and collection steps were repeated. The protocol was then followed per manufacturer's instructions, including the DNase 1 treatment step. The final elution volume was 120 μ l. RNA was quantified using the Quant-iT RiboGreen RNA Reagent and Kit (Invitrogen) and a Turner Biosystems Spectrophotometer. Approximately 30 ng of total RNA was reverse transcribed using the High Capacity RNA-to-cDNA Kit (Applied Biosystems) according to the manufacturer's instructions.

### soxB gene primer design and PCR optimization using sulfurimonas denitrificans

Primers were designed using multiple alignments of *soxB* genes from Epsilonproteobacterial species *Nitratiruptor* sp. SB155-2, *Sulfurovum* sp. NBC-37, and *Sulfurimonas denitrificans* DSM 1225 (Table 2). Initial primer sequences were created using the *soxB* nucleotide sequences in Primaclade (Gadberry et al., 2005) and PriFi (Fredslund et al., 2005), and the amino acid sequences in iCODEHOP (Boyce et al., 2009). Sequences were then manually adjusted based on the initial bacterial sequences. CODEHOPs are hybrid primers that consist of a 5′ nondegenerate clamp, followed by a 3′ degenerate core sequence (indicated in lowercase nucleotides) that stabilizes hybridization (Rose, 2003). Primers were manufactured by Invitrogen (Carlsbad, CA).

Table 2**soxB primers designed and PCR settings used in this study**.

**Table:** 

**Primer**	**Sequence**	**Target nucleotide position^a^**
sox190F	5′-TGGAGRGAGCCWTCAAC-3′	190–206
sox527F	5′-TGGTWGGWCAYTGGGAATTTA-3′	527–547
sox523F	5′-GTGATGGTTGGACAytgggartwya-3′	523–547
sox1198R	5′-AGAANGTATCTCKYTTATAAAG-3′	1198–1177
sox1210R	5′-CGAAGGTGGAGTAGAAngtrtctckytt-3′	1210–1183
sox1292R	5′-GTCGTTCCCCATckrtanccngg-3′	1292–1270
F-34R	5′-CTCAAAGGTGTAAACGtynggatakgt-3′

**Table:** 

**Primer combinations**	**Annealing temperature (°C)**	**Amplicon length (bp)**
sox190F/sox1198R	45	1009
sox527F/sox1198R	46	672
sox523F/sox1210R	52	688
sox523F/sox1292R	52	770

a Using S. denitrificans numbering.

#### Sulfurimonas denitrificans

DSM 1251 was obtained from the DSMZ culture collection and grown using DSMZ Medium 113, with an additional 10 g/L NaCl and substituting trace element solution 141 (from DSMZ Medium 141) for trace element solution SL-4. DNA was extracted from *S. denitrificans* using the UltraClean Microbial DNA Isolation Kit (MoBio Laboratories, Inc.), using 3 mL of culture and an initial extended centrifugation time (8 min).

Optimal annealing conditions for the primers were determined via gradient PCR using *S. denitrificans* DNA as template. Primers were tested in the following pairs (Table 2): sox190F/sox1198R, sox527F/sox1198R, sox523F/sox1210R, sox523F/sox1292R, and sox523F/F-34R. Gradients were 42–51°C for sox190F/sox1198R, and 52–60°C for all other primer sets. Annealing temperatures of 45°C for sox190F/sox1198R, 46°C for sox527F/sox1198R, and 52°C for sox523F/sox1210R and sox523F/sox1292R were chosen based on visual inspection of the gradient PCR results on a 1% agarose gel stained with ethidium bromide and photographed under UV light. The PCR reaction mixture consisted of 1X buffer (Promega), 4 mM MgCl_2_, 0.2 mM of each deoxynucleoside triphosphate (dNTP), 0.6 μ M of each primer, 1U GoTaq polymerase (Promega), 1 μ L DNA template, and DEPC H_2_O to 25 μ L. Thermocycling conditions on an Eppendorf thermal cycler consisted of an initial denaturation step at 94°C for 3 min, followed by 35 cycles at 94°C for 30 s, applicable annealing temperature for 45 s, and 72°C for 1 min, followed by a final extension at 72°C for 5 min. Using these PCR conditions, all primer sets yielded a single band amplicon of the predicted size, which ranged from ~670 to 1000 bp depending on the primer set. PCR products were verified to be the correct size on a 1% agarose gel stained with ethidium bromide and photographed under UV light. PCR products were cleaned using the MinElute PCR Purification Kit (Qiagen) following the manufacturer's instructions and directly sequenced on an Applied Biosystems 3730XL sequencer. DNA sequences were compared to sequences in the NCBI database via BLAST and in all cases were the *soxB* gene of *S. denitrificans* DSM 1251.

### soxB gene cloning, sequencing, and analysis

Each of the 4 primer sets were used with appropriate PCR settings as outlined above on DNA and cDNA from all fluid samples. PCR was visualized by UV light on ethidium bromide-stained 1% agarose gels. While multiple primer sets were tested on each sample, only amplicons from primer pair sox527F/1198R were cloned and sequenced. For the background seawater sample, only primer pair sox 523F/1292R amplified, thus it was cloned and sequenced. PCR products were purified using the MinElute PCR Purification Kit (Qiagen) and the products analyzed on 0.8% agarose gel. Products were gel excised, purified, cloned, and sequenced bi-directionally as described in Huber et al. (2009). Primer sequences were trimmed from nucleotide sequences and translated into amino acids using EMBOSS Transeq (Rice et al., 2000). A protein distance matrix was calculated in ProtDist (Felsenstein, 1989) and operational taxonomic units (OTUs) constructed in mothur (Schloss et al., 2009). Phylogenetic relationships of representative 85% amino acid identity OTUs to other *soxB* genes were determined using MEGA5 (Tamura et al., 2007). Sequences are deposited in GenBank under the following Accession Numbers: KC793341-KC793868.

### 454 pyrosequencing and analysis

Amplicons targeting the V4V6 rRNA hypervariable region were generated for each DNA and cDNA sample following Filkins et al. (2012). A custom bioinformatic pipeline was used to remove low quality reads according to Filkins et al. (2012). The algorithm GAST assigned taxonomy to each unique read (Huse et al., 2008). All high quality reads that passed initial processing were further analyzed and trimmed to the V5 region based on high error rates at the V4 end, resulting in ~350 bp reads, and UCLUST (Edgar, 2010) identified operational taxonomic units (OTUs) with 96% sequence identity. Singletons were removed from all subsequent reported analyses. The resulting matrix of OTUs shared between samples was used to construct taxonomic summaries and compare background seawater and vent samples. The OTU matrices were normalized to total and transformed via square root and used to calculate a distance matrix with the Morisita–Horn measure (Horn, 1966). Distance matrices were imported into PRIMER-E (Clarke and Gorley, 2006) for a variety of analyses, including hierarchical cluster analysis, principal component analysis (PCO), analysis of similarity (ANOSIM), and nonmetric multidimensional scaling (MDS) analysis. The entire chemical dataset was also included for PCA and Spearman correlation analyses. To predict which OTUs were active or inactive, the top OTUs that occurred greater than 0.1% of the total sequences in either the RNA or DNA fraction for both the *Epsilon*- and *Gammaproteobacteria* were examined. Each OTU was scored as active or inactive based on its recovery in DNA and RNA samples (Jones and Lennon, 2010). Data is accessible via vamps.mbl.edu under project name JAH_AXV_Bv6v4.

## Results

### Axial seamount fluid samples

Fluid samples were collected from basalt- or sulfide-hosted diffuse flow venting sites with varying physical and chemical parameters, as well as from background seawater (Table 1). Gollum, Marker 33, and Marker 113 sites discharge vent fluids directly from basalt, while Pompeii, Escargot, and 9 m sites discharge vent fluids from the sides of metal-sulfide chimneys. Fluids at Pompeii were emanating from a large clump of tubeworms. These samples span a range of sulfide to heat ratios (calculated using the measured H_2_S concentration and the average in-line temperature of individual samples, Table 1), reflecting different concentrations of hydrogen sulfide in end-member hydrothermal fluids and variable sulfide oxidation (Butterfield et al., 2004). The site with the lowest sulfide/heat ratio of 1.0 nmol/J was Gollum, the mid-range site Pompeii had a ratio of 4, and the other four sample sites had ratios between 6 and 9. Average temperatures ranged from 20.6 to 49.7°C. The measured temperatures and magnesium content of the collected samples indicate that the fluids are 79–96% seawater (4–21% hot hydrothermal end-member) overall, but this percentage does not tell us their mixing history or subseafloor residence time.

### soxB gene data

Primers that specifically target the *Epsilonproteobacteria soxB* gene were designed by comparing conserved regions of the *Sulfurimonas denitrificans* DSM 1225, *Sulfurovum* sp. NBC37-1, and *Nitratiruptor* sp. SB155-2 *soxB* genes (Nakagawa et al., 2007; Sievert et al., 2008b). The primers are not capable of identifying *Arcobacter*-like sequences. The *Arcobacter soxB* sequences cluster in a different clade (Hügler et al., 2010), and homologous regions for primer annealing could not be identified across all *Epsilonproteobacteria* genera, while excluding other groups. The primers were tested and PCR settings were optimized using DNA extracted from a pure culture of *S. denitrificans*. Three forward and three reverse primers were used in four combinations (Table 2). The sequences were translated to amino acid residues and checked via BLASTP and alignment to verify amplification of the *soxB* gene. The four different primer sets amplified *soxB* gene fragments ranging in size from 672 to 1009 bp. The primer sets were additionally tested on environmental DNA samples and were shown to be capable of amplifying *Sulfurovum*-, *Sulfurimonas*-, and *Nitratiruptor*-like *soxB* sequences (Meyer et al., 2013).

The four *soxB* primer sets were used to amplify soxB from the DNA and RNA fractions of the six Axial Seamount hydrothermal vent fluid samples and the background seawater sample. Positive amplicons were obtained from six of the seven DNA fractions, with the exception of Pompeii. In the RNA fractions, amplicons were not obtained from Pompeii, 9 m, and the background seawater sample. A total of 480 clones were sequenced across the nine DNA and RNA vent fluid samples and the one DNA background seawater sample (Table 3).

**Table 3:** **Sequencing statistics for 16S rRNA and *soxB* gene sequences**.

	**Nucleic acid**	**Total tag sequences^a^**	**Total epsilon tag sequences**	**% Epsilons**	**Total soxB sequences^b^**
Gollum	DNA	14866	7000	47.1	47
	RNA	11128	157	1.4	48
Marker 33	DNA	15773	8117	51.5	45
	RNA	11166	248	2.2	45
Marker 113	DNA	14820	3998	27.0	38
	RNA	14451	2870	19.9	46
Pompeii	DNA	16538	6699	40.5	–
	RNA	10475	694	6.6	–
Escargot	DNA	15457	6174	39.9	46
	RNA	98143	18453	18.8	46
9 m	DNA	17570	3241	18.4	44
	RNA	10256	407	4.0	–
Seawater	DNA	11682	0	0	75
Total	–	262325	58058	–	480

a No singletons.

b Primer set 527F/1198R was used for all vent samples; 523F/1292 for seawater.

**Figure 1:**
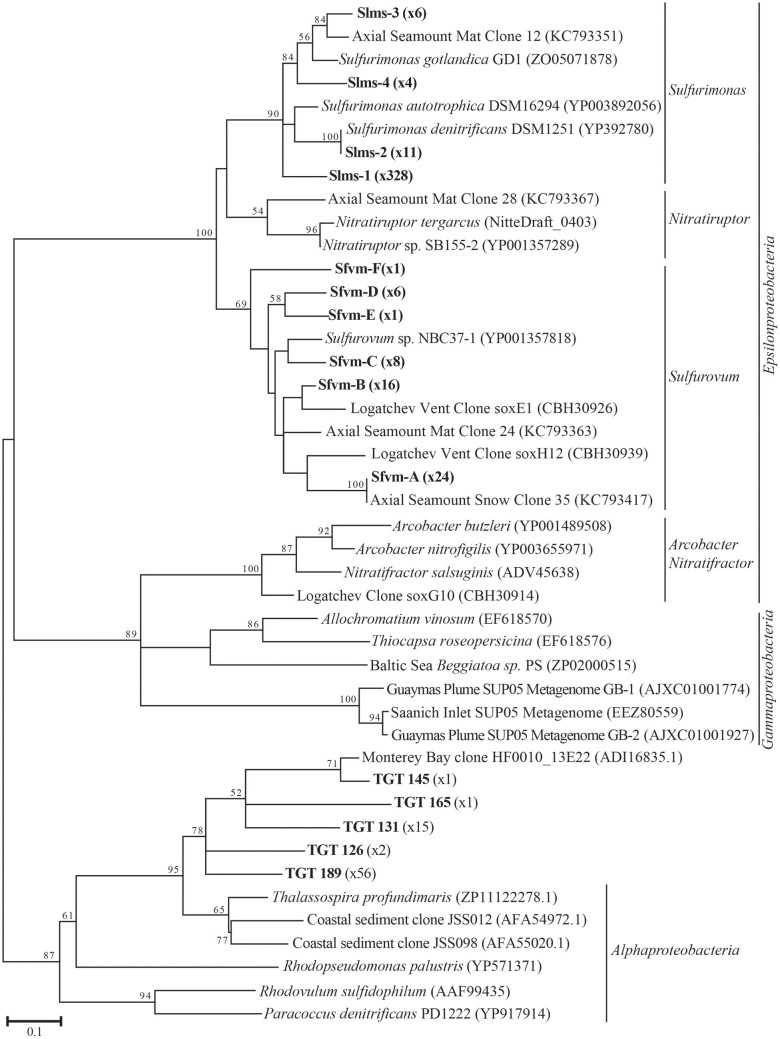
**Maximum likelihood phylogenetic tree showing the relationships of the 15% *soxB* gene OTUs found in this study (indicated in boldface) with number of sequences belonging to each OTU indicated in parentheses**. The maximum likelihood tree was calculated based on ~234 amino acid residues and bootstrap values above 50 are shown.

**Figure 2:**
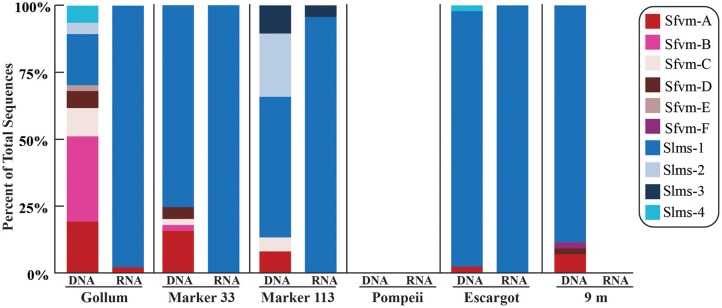
**Relative abundance with taxonomic affiliation of the 15% *soxB* gene OTUs in the DNA and RNA fractions of different vents classified according to the phylogenetic tree in Figure 1**.

All hydrothermal vent *soxB* clones were most closely related to the *Sulfurimonas* (Slms) and *Sulfurovum* (Sfvm) genera in the *Epsilonproteobacteria* and represented 10 different phylotypes at an 85% amino acid identity level (Figures 1, 2). Of the 405 clones from the nine hydrothermal libraries, all but 37 fell into 3 main OTUs. The most common OTU, Slms-1, represented ~80% of all the clones and was detected at all positively amplified hydrothermal samples in both the DNA and RNA fractions. The other two most common clone sequences were *Sulfurovum*-like phylotypes Sfvm-A and Sfvm-B, which represented 6 and 4%, respectively, of all hydrothermal clones. Sfvm-A was detected in the DNA fraction of all sites, but only in the RNA fraction at Gollum. Sfvm-B was only detected in the DNA fractions at Gollum and Marker 33 and in none of the RNA fractions (Figure 2).

Overall, only one *Sulfurovum*-like *soxB* gene was found in the RNA fraction (Gollum). Instead, Slms-1 was the only *soxB* gene detected in all of the RNA fractions (Figure 2) and was also the most abundant *soxB* gene in all DNA fractions, except for Gollum. At Gollum, the composition of the DNA and RNA libraries differed, with the DNA library dominated by *Sulfurovum*-like *soxB* gene sequences and the RNA fraction almost completely composed of Slms-1. No comparisons could be made between the DNA and RNA fractions of the 9 m site since *soxB* was not amplified from the RNA fraction.

In the background seawater sample, a total of five phylotypes were found, with the two most dominant OTUs representing 94% of the background clones. All phylotypes were most closely related to *Alphaproteobacteria*, although these BLASTP matches were made at approximately 67% identity rather than the 90–99% identity of the *Epsilonproteobacteria* BLASTP hits in the libraries from the venting fluids (Figure 1). These *Alphaprotoebacteria*-like sequences were not found in the vent samples.

### 16S rRNA gene 454 pyrosequencing

**Figure 3:**
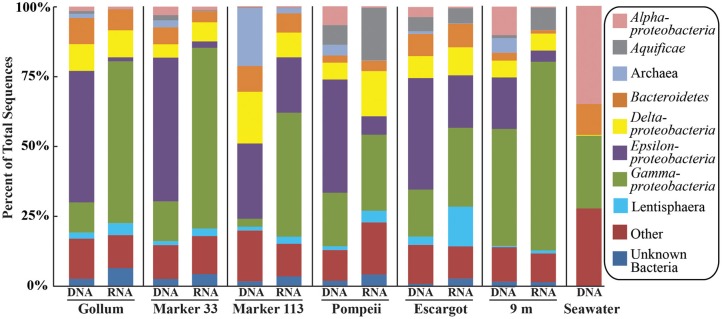
**Taxonomic breakdown and relative abundance at the phylum (and class for Proteobacteria) level for the 96% 16S rRNA gene OTUs in all samples**. Only those taxa that occurred more than 3% in any individual dataset are included. Taxa that occurred less than 3% are placed into “Other.”

**Figure 4:**
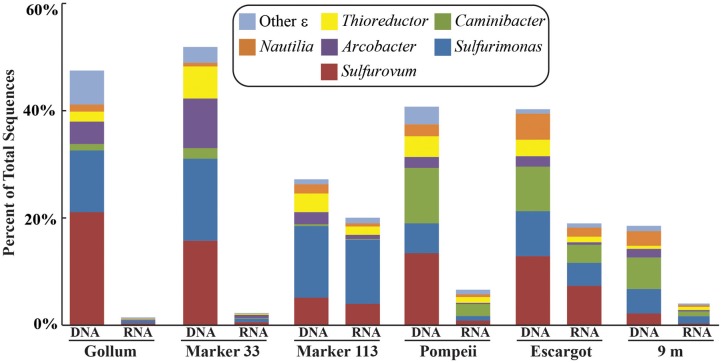
**Taxonomic breakdown and relative abundance of dominant *Epsilonproteobacteria* at the genus level for the 96% 16S rRNA gene OTUs in each vent fluid sample**. All other Epsilonproteobacterial sequences are placed into “Other.”

454 pyrosequencing of the bacterial 16S rRNA gene from both the DNA and cDNA from all samples resulted in 262,325 high-quality non-singleton tag sequences across the hydrothermal and background seawater sites (a DNA library only was constructed for the background seawater sample, Table 3). *Epsilonproteobacteria* (58,058 sequences) were present in all 12 hydrothermal vent fluid libraries (Table 3, Figure 3). Epsilonproteobacterial sequences were not detected in background seawater, even before singletons were removed (Table 3, Figure 3). The background seawater sample was mostly composed of *Alpha*- and *Gammaproteobacteria* (Figure 3). Within the *Epsilonproteobacteria*, sequences of the genera *Sulfurimonas and Sulfurovum* dominated most libraries (Figure 4), although at Pompeii, Escargot, and 9 m, numerous *Caminibacter* sequences were also detected (Figure 4). The only other major group within the *Epsilonproteobacteria* was *Arcobacter*, which comprised almost 9% of the DNA fraction at Marker 33. In all cases, *Epsilonproteobacteria* sequences were more abundant in DNA fractions than RNA fractions for each individual vent (Figure 4). Comparison of rRNA profiles of the most abundant OTUs for *Epsilonproteobacteria* shows most *Epsilonproteobacteria* were inactive in the sampled vent fluids. Out of 164 *Epsilonproteobacteria* OTUs considered, only 22 were scored as active (Table 4).

**Table 4:** **Predicted percent activity based on 16S rRNA gene data**.

	***Epsilonproteobacteria***			***Gammaproteobacteria***		
**Site**	**Total OTUs^a^**	**Active OTUs^b^**	**% Active**	**Total OTUs^a^**	**Active OTUs^b^**	**% Active**
Gollum	94	0	0	25	18	72
Marker 33	79	0	0	39	35	89.7
Marker 113	41	17	41.5	13	13	100
Pompeii	47	0	0	14	10	71.4
Escargot	61	2	3.3	18	11	61.1
9m	37	3	8.1	19	9	47.4

a Number of OTUs that occur >0.1% of all sequences in either RNA or DNA fraction.

b Number of OTUs that were scored as active if the RNA relative recovery was greater than its recovery from DNA.

**Figure 5:**
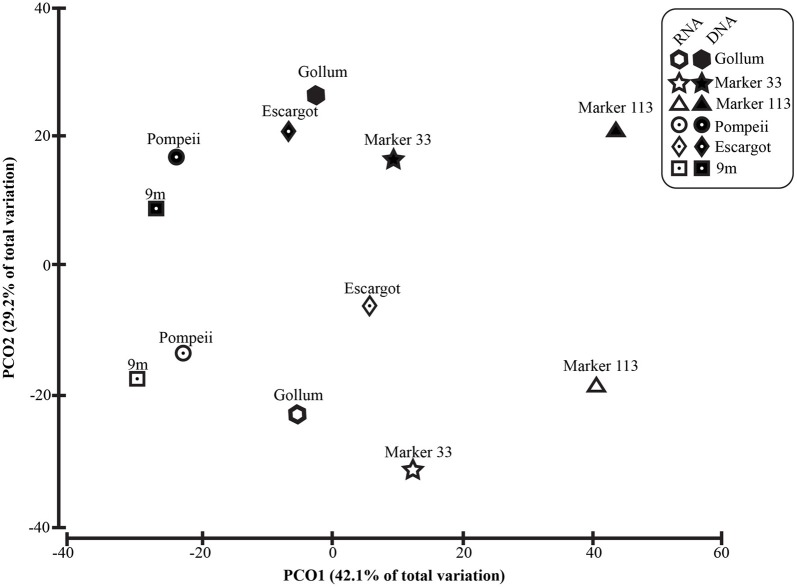
**Principal coordinates analysis of all 96% 16S rRNA gene OTUs**.

**Figure 6:**
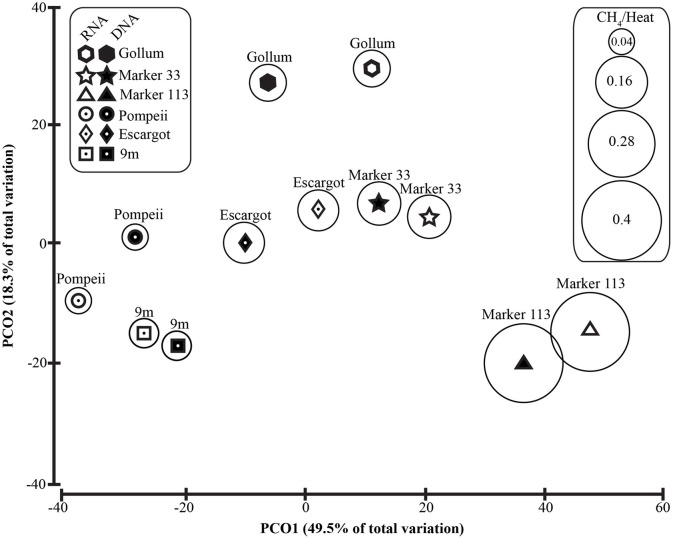
**Principal coordinates analysis of all 96% Epsilonproteobacterial 16S rRNA gene OTUs with CH_4_/Heat ratios shown in bubbles surrounding symbols**.

To distinguish vent-specific microbial communities from background seawater communities that mix with venting fluids at the seafloor, all 16S rRNA gene OTUs as well as only Epsilonproteobacterial 16S rRNA gene OTUs were analyzed separately. SIMPROF tests of all OTU datasets indicated there is significant community structure among the individual samples (π = 6.48, *P* value < 0.001 for all OTUs. π = 8.46, *P* value < 0.001 for Epsilonprotoebacterial OTUs). One-Way ANOSIM (analysis of similarity) tests with a variety of factors resulted in significant global R values for individual vent sampled and type of vent (basalt or sulfide) for both all OTUs and only Epsilonprotebacterial OTUs; values were higher for *Epsilonproteobacteria* (*R* = 0.84, *P* value < 0.001 for *Epsilonproteobacteria* and individual vent; *R* = 0.55, *P* value = 0.004 for *Epsilonproteobacteria* and type of vent). The type of nucleic acid analyzed (RNA or DNA) was significant only when all OTUs were considered (*R* = 0.39, *P* value = 0.006). Principal coordinates analysis of all OTUs showed a clear distinction between DNA and RNA fractions (Figure 5) that was not seen when only Epsilonproteobacterial OTUs were analyzed in the same manner (Figure 6). BEST analysis [Biota and/or Environmental matching (Clarke and Gorley, 2006)] was used to match multivariate patterns of OTUs to chemical variables measured in the vent fluids (Table 1). For both all OTUs and only Epsilonproteobacterial OTUs, the most significant R value was with CH_4_/Heat (*R* = 0.69, *P* value = 0.003, Figure 6).

**Figure 7:**
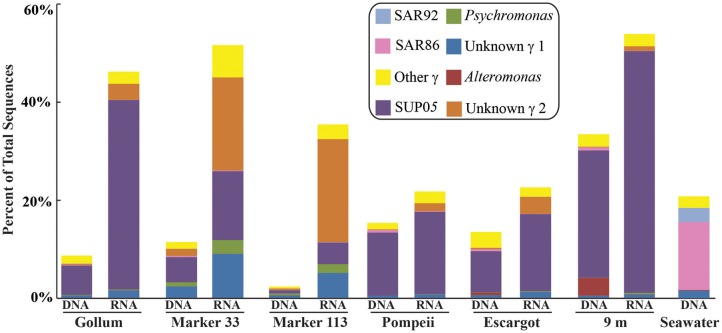
**Taxonomic breakdown and relative abundance of dominant *Gammaproteobacteria* at the genus level for 96% 16S rRNA gene OTUs in each sample**. All other *Gammaproteobacteria* sequences are lumped into “Other.”

Gammaproteobacterial sequences were also treated separately due to their relatively high abundance in all RNA fractions (Figure 3). They were detected in all 12 hydrothermal vent fluid libraries, as well as background seawater. Within the *Gammaproteobacteria*, sequences belonging to the SUP05 clade were most frequently recovered, as well as those sequences related to animal endo- and ecto-symbionts (Figure 7, labeled “Unknown γ 2”). Principal coordinates analysis of all Gammaproteobacterial OTUs showed a clear distinction between DNA and RNA fractions (data not shown), similar to that seen for all OTUs. Gammaproteobacterial sequences were more abundant in RNA fractions than DNA fractions (Figure 7). Comparison of RNA and DNA profiles of the most abundant OTUs for *Gammaproteobacteria* shows high activity of *Gammaproteobacteri*a in most vent fluids. Out of 67 *Gammaproteobacteria* OTUs considered, 56 were scored as active (Table 4).

## Discussion

Low-temperature diffuse vent samples represent combined hot hydrothermal vent fluid and deep seawater that mix and react beneath the seafloor. Mixing begins beneath the seafloor and continues as fluids vent and are diluted by ambient seawater as they rise up into the water column. By controlled pumping of fluids into a sampler intake that is inserted into the vents, the mixing that occurs with ambient seawater is controlled. However, even if dilution of the vent fluids with ambient seawater during sampling is not completely avoided, it is both possible and likely that seawater is entrained into the fluid along shallow mixing pathways in the subseafloor. Similarly, the microbial composition of the fluids reflects both seawater and subseafloor communities, which means that detection of vent-specific microbial processes can be challenging. By removing sequences from seawater organisms detected in background seawater from the vent fluid sample sequences, the analysis can focus on the diversity, distribution, and gene expression of vent-endemic subseafloor microbes. For this study, *Epsilonproteobacteria* that were not detected in background seawater were targeted to analyze their diversity, distribution, and sulfur-oxidizing gene patterns in the subseafloor.

The *soxB* gene primers were designed to screen for the hydrothermal vent sulfur-oxidizing *Epsilonproteobacteria* belonging to the genera *Sulfurimonas*, *Sulfurovum*, and *Nitratiruptor*. In particular, *Sulfurimonas* and *Sulfurimonas* are frequently detected at high abundances in diverse marine environments where strong gradients of oxygen and sulfide exist, including cold seep sediments (Roalkvam et al., 2011), waters of redoxclines (Grote et al., 2007), coastal marine sediments (Hoor, 1975), and both shallow and deep hydrothermal vents (Huber et al., 2010; Zhang et al., 2012). The primers do not detect *Arcobacter*, a genus within the *Epsilonproteobacteria* that also uses the Sox pathway. The 454 16S rRNA gene data in this study suggests *Arcobacter* are not dominant in most hydrothermal vent fluids at Axial Seamount, and while they are sometimes seen in high abundance (Huber et al., 2007), a single primer set to capture all *Epsilonprotebacteria* that use the Sox pathway was not possible to design due to the distant relationship in the *soxB* gene between the different genera (Meyer and Kuever, 2007; Headd and Engel, 2013).

The *soxB* gene OTUs observed as *Sulfurovum*-like in this study generally shared the most identity with *Sulfurovum* sp. NBC37-1, which was isolated from a deep-sea hydrothermal vent and is capable of both hydrogen and sulfur oxidation, as well as denitrification (Nakagawa et al., 2007; Yamamoto et al., 2010). Two other members of this genus have been isolated; one from a hydrothermal vent (*S. lithotrophicum*) and one from marine sediments (Candidatus *S. sediminum*). Both are also described as sulfur oxidizers, although neither has been shown to be capable of hydrogen utilization like NBC37-1 (Inagaki et al., 2004; Park et al., 2012). The *soxB* gene OTUs observed as *Sulfurimonas*-like in this study were closely related to cultivated species of *Sulfurimonas*. *Sulfurimonas autotrophica* was originally isolated from hydrothermal sediments (Inagaki et al., 2003), and other members of the genus have been identified associated with vent animals (Takai et al., 2006), in the redoxcline of the Baltic Sea (Grote et al., 2012), and in coastal marine sediments (Hoor, 1975; Sievert et al., 2008b). Genomic sequencing of some of these isolates shows multiple functional genes for different metabolic pathways such as sulfur oxidation, nitrate reduction, and hydrogen oxidation, highlighting their metabolic flexibility and similarity to members of the *Sulfurovum* (Sievert et al., 2008a; Grote et al., 2012). A key trait of all known isolates of both genera is that they cannot grow at 100% oxygen saturation; they are all described as facultative anaerobes (Inagaki et al., 2004; Takai et al., 2006). These two closely-related genera of *Epsilonproteobacteria* are likely occupying the same subseafloor habitat, but the *soxB* gene data suggests dominance of *Sulfurimonas* in active sulfur oxidation via the Sox pathway.

The *soxB* gene was not successfully amplified from either the DNA or RNA fraction of the Pompeii site. 16S rRNA profiles show *Sulfurimonas* and *Sulfurovum* are present at this site (Figure 4), and the geochemical conditions are within the range of growth of these organisms, therefore it is not clear why soxB did not amplify from these samples. In addition, the examination of *Sulfurimonas* and *Sulfurovum* 16S rRNA gene OTUs did not reveal any patterns specific to these populations at Pompeii and the reason for the lack of soxB detection at this site remains unknown.

At 9 m, the *soxB* gene was not amplified from the RNA fraction, although soxB was detected in the DNA library. This is likely attributed to the high temperature (maximum 51.3°C) of this metal-sulfide chimney site. Above ~40°C, sulfur oxidation is not favored due to severe oxygen limitation (McCollom and Shock, 1997; Butterfield et al., 2004), and known growth optima of all known cultures within *Sulfurimonas* and *Sulfurovum* are below 40°C (Inagaki et al., 2004; Takai et al., 2006). This site also had the lowest pH (4.8), which is still within the range of growth for *Sulfurovum lithotrophicum* (Inagaki et al., 2004), but outside of the range of growth for *Sulfurimonas paralvinellae* (Takai et al., 2006). A combination of low pH and high temperature likely contributes to the lack of *soxB* gene transcripts detected at this site.

At Gollum, the *soxB* genes and gene transcripts from the DNA and RNA fractions had very different signatures, with diverse *Sulfurovum*-like OTUs dominating the DNA fraction and a single *Sulfurimonas*-like OTU comprising almost the entire RNA fraction. In fact, the RNA profiles of *soxB* gene transcripts were nearly identical among the four vent fluid samples where soxB amplified and suggest that *Sulfurimonas*-like species are out-competing *Sulfurovum* in actively oxidizing sulfur via the Sox pathway in the subseafloor beneath each vent (Figure 2). Significant correlation between sulfide and proportions of *Sulfurovum* has previously been seen in diffuse fluids based on 16S rRNA gene sequencing (Perner et al., 2013), but here there were no statistically significant correlations in this genus with chemistry from either the 454 16S rRNA or *soxB* gene data. However, Gollum has the lowest concentration of hydrogen sulfide (84 μM, Table 1), as well as the lowest H_2_S/Heat ratio, suggesting low sulfide levels may favor the presence of *Sulfurovum* carrying the *soxB* gene. But given that all RNA profiles are dominated by Slms-1, there does not appear to be any correlation with respect to expression of *soxB* genes and sulfide to heat ratios or any other chemical variables.

Unlike recent work in terrestrial springs on sulfur oxidation genes (Headd and Engel, 2013), there is no evidence of niche partitioning in the active communities of *Epsilonproteobacteria* expressing the *soxB* gene. However, when examining all of the *Epsilonproteobacteria* 16S rRNA gene sequences (Figure 6), there was significant groupings related to the individual vent sampled and the type of vent (basalt or sulfide), highlighting the importance of physical isolation in helping to maintain stable microbial communities in the subseafloor (Huber et al., 2006, 2010; Opatkiewicz et al., 2009). There were also significant groupings of samples that correlated with chemical variables. The most significant correlation was with methane. Those vents high in methane are often conversely low in hydrogen (Figure 6, Table 1), suggesting hydrogen is being drawn down and methane generated by hydrogen-utilizing methanogens (Butterfield et al., 2004; Ver Eecke et al., 2012). At Marker 113, where the highest methane concentrations were seen, methanogenic archaea belonging to the genera *Methanocaldococcus, Methanococcus*, and *Methanothermococcus* were detected with the universal bacterial primer set, consistent with previous work at Marker 113 that showed hydrogenotrophic methanogens are abundant and active at this site (Ver Eecke et al., 2012).

There are no known *Epsilonproteobacteria* that use or generate methane, but many use hydrogen as an electron donor, including *Caminibacter* and *Nautilia* within the *Nautiliaceae* (Campbell et al., 2006). A higher relative abundance of tag sequences corresponding to these two genera were detected at Pompeii, Escargot, and 9 m, and these samples were all collected from the sides of sulfide chimneys that had among the highest hydrogen concentrations (Table 1, Figure 4), suggesting the combination of geological structure and chemistry may favor these thermophilic, hydrogen-utilizing, sulfur-reducing *Epsilonproteobacteria*. This probably reflects the different niches these organisms occupy. *Sulfurimonas* and *Sulfurovum* are likely living in the more shallow, cool subseafloor where microaerobic niches exist, whereas members of the *Nautiliaceae* are likely residing further beneath the seafloor or in walls of sulfide chimneys in warm, anaerobic niches with enriched hydrogen. However, despite the overall structuring of the Epsilonproteobacterial microbial community revealed by 16S rRNA gene sequencing, no patterns of the functional *soxB* gene were seen, suggesting that different lineages at each vent may be carrying out similar metabolic functions.

In addition to examining community patterns and gene expression of subseafloor *Epsilonproteobacteria*, the side-by-side comparison of 16S rRNA gene patterns from DNA and RNA revealed that *Gammaproteobacteria* were present and active at all Axial Seamount vent sites (Table 4). The most abundant and active OTU belonged to the SUP05 clade, a ubiquitous group of bacteria frequently found in symbiotic association with hydrothermal vent animals (Newton et al., 2007) as well as in hydrothermal plumes, non-plume deep-sea water (Sunamura et al., 2004; Anantharaman et al., 2013; Dick et al., 2013), and oxygen minimum zones (Lavik et al., 2009; Walsh et al., 2009). Recent quantitative PCR data in low-temperature diffuse vents from Axial Seamount and Endeavour Segment also indicates SUP05 constitute up to 38% of all bacteria in some vent fluids (Bourbonnais et al., 2012; Anderson et al., 2013). There are no SUP05 from the deep sea in culture, but metagenomic and metatranscriptomic datasets indicate they are obligate autotrophs capable of sulfur and hydrogen oxidation, nitrate reduction, as well as storage of sulfur in globules (Walsh et al., 2009; Wright et al., 2012; Anantharaman et al., 2013). Active transcripts of multiple sulfur oxidation genes have even been detected in background seawater (Anantharaman et al., 2013), and while one metagenomic study suggests they are facultative anaerobes or strict anaerobes (Walsh et al., 2009), another suggests they can use oxygen in both fully aerobic and microaerobic conditions (Anantharaman et al., 2013).

There were a small number of sequences belonging to the SUP05 clade in the background seawater sample, and they were highly enriched in vent fluid samples, particularly the RNA fractions. However, when the Gammaproteobacterial 16S rRNA gene OTUs were analyzed, there were similar patterns to those seen when all OTUs were considered (Figure 5). This suggests that SUP05 are not vent-endemic or living in the subseafloor, but instead originate from background seawater and are enriched where the oxygen-rich deep seawater mixes with hydrogen sulfide-rich vent fluids in the very shallow subseafloor or as they exit the seafloor at the point of sampling. In contrast, the *Epsilonproteobacteria* sampled are being entrained into vent fluids from beneath the seafloor, where microaerobic and anaerobic pockets exist. Because the samples are being fixed immediately after filtration, those organisms active closer to the point of sampling will be over-represented in the RNA fraction. Organisms abundant within the seafloor will also be captured, but their activity will likely not be as representative of their *in situ* activity, given the unknown length of time for fluids and their resident microbes to travel from their particular subseafloor niche to the seafloor.

Many challenges remain in elucidating the subseafloor microbial ecosystem, but the coupled phylogenetic and functional gene analysis of DNA and RNA from low temperature diffuse vent fluids allows for a fuller picture of what may be occurring both at and within the seafloor at deep-sea hydrothermal vents. The detection of *soxB* gene transcripts shows there are active sulfur-oxidizing *Epsilonproteobacteria* within the subseafloor at Axial Seamount, mostly belonging to the genus *Sulfurimonas.* The 16S rRNA data suggests SUP05 are extremely active in the shallow subseafloor or at the seafloor where fluids are exiting from the crust and mixing with background seawater, the likely habitat of these organisms. Within the seafloor, it is predicted that *Epsilonproteobacteria* are the main sulfur oxidizers, given their ability to tolerate very low oxygen concentrations and use sulfur compounds as both electron acceptors and donors (Inagaki et al., 2004; Yamamoto and Takai, 2011). These results are consistent with previous work by Yamamoto and Takai (2011) and Anderson et al. (2013) suggesting that *Gammaproteobacteria* dominate when both oxygen and reduced sulfur compounds are steadily supplied but once oxygen begins to disappear, *Epsilonproteobacteria* have greater physiological and metabolic flexibility and potential to occupy diverse vent niches, especially in the subseafloor. Targeted functional approaches that focus on activity and expression of particular metabolic pathways, such as the primers developed here combined with incubation experiments or *in situ* studies, will help elucidate how these organisms differentiate into their respective niches and contribute to the biogeochemical cycling of sulfur and carbon both in the deep sea and subseafloor.

### Conflict of interest statement

The authors declare that the research was conducted in the absence of any commercial or financial relationships that could be construed as a potential conflict of interest.
